# Leisure screen time and diabetic retinopathy risk: A Mendelian randomization study

**DOI:** 10.1097/MD.0000000000040099

**Published:** 2024-10-25

**Authors:** Yuan-Yuan Tang, Jun-Jie Liu, Hong-Jing Gu, Xiao-Shu Wang, Chun-Mei Tan

**Affiliations:** a Department of Nephroendocrinology, Guang’an People’s Hospital, Guan’an, Sichuan, China.

**Keywords:** diabetic retinopathy, leisure screen time, Mendelian randomization

## Abstract

The aim of this study was to investigate whether leisure screen time (LST) increases the risk of diabetic retinopathy (DR) using the Mendelian randomization (MR). This study employed a two-sample MR analysis, utilizing 63 single-nucleotide polymorphisms as instrumental variables (IVs) to assess the causal relationship between LST and the risk of Dr. To ensure the robustness of the results, a multi-effect test was conducted to evaluate the validity of the IVs. Additionally, heterogeneity tests were performed to explore differences among sub-samples. Sensitivity analyses were also conducted to further validate our findings. The impact of LST on the risk of DR was observed in both inverse variance weighted (odds ratio [OR]: 1.22, 95% confidence interval [CI]: 1.04–1.43, *P* = 1.38 × 10^‐2^) and weighted median (OR: 1.30, 95% CI: 1.05–1.61, *P* = 1.46 × 10^‐2^) analyses. However, the MR-Egger method (OR: 0.66, 95% CI: 0.32–1.36, *P* = .273) did not find an increased risk of DR with increased LST. The pleiotropy test yielded a *P*-value of *P* = .09. Heterogeneity tests showed that the *Q* value for the inverse variance weighted method was 71.39 with a *P*-value of 0.17, indicating no significant heterogeneity. These results suggest that the IVs might be appropriate, and the analysis results could be robust. A large-scale MR analysis suggests a causal relationship between LST and the risk of Dr.

## 1. Introduction

Diabetic retinopathy (DR) is a typical microvascular complication caused by diabetes, and is a leading cause of blindness in adults, associated with an increased risk of life-threatening systemic vascular complications.^[1]^ Currently, about 10% of patients with DR are in the potential stage of vision loss.^[2]^ Therefore, it is crucial to identify the risk factors for DR in order to develop and implement effective intervention strategies, not only to slow down the progression of DR but also to safeguard the quality of life and visual health of diabetic patients.

With the rapid development of information technology, the increase in screen time, especially leisure screen time (LST), has become a prominent feature of modern life. LST typically refers to the time individuals spend using electronic devices (such as smartphones, computers, tablets, and televisions) outside of work or study. Research has shown that the lack of physical activity has significant effects on the development of various chronic diseases, including cardiovascular diseases, diabetic nephropathy, and various types of cancer.^[3]^ The increase in sedentary behavior, accompanied by a general decrease in physical activity levels, may pose an independent threat to public health.^[4]^

Sedentary behavior has been identified as a modifiable risk factor for diabetes.^[5–7]^ However, little is known about the association between LST (a main sedentary activity) and Dr. The development of DR is associated with prolonged diabetes duration, elevated blood pressure, and poor glycemic control. However, these factors can only partially explain the risk of retinopathy in diabetic patients. A study showed that television viewing time was independently associated with abnormal retinal vascular signs.^[8]^ Another study suggested that sedentary behavior assessed by accelerometry was a risk factor for DR.^[9]^ Due to the potential methodological limitations of observational studies, the causal relationship between these factors and DR may be confounded by reverse causality or by race, gender, and age.

Mendelian randomization (MR) is a method that uses genetic variation as instrumental variables (IVs) to assess causal relationships between exposure and disease, which can overcome some limitations of traditional observational studies.^[10]^ Using this method, we can more accurately assess whether LST is an independent risk factor for Dr.

This study aims to explore whether LST increases the risk of DR through a two-sample MR analysis using genetic variation as IVs. This study not only provides evidence for understanding the potential causal relationship between LST and DR but also may offer new lifestyle intervention strategies for the prevention of Dr.

## 2. Materials and methods

### 2.1. Ethics statement

The summary-level data used in this study are de-identified public data and are available for download. Each genome-wide association study (GWAS) involved in this study was ethically approved by the respective institutions.

### 2.2. Overall study design

**Figure 1.**
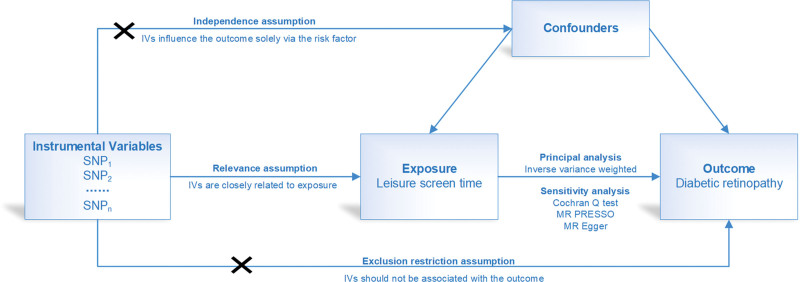
The overview of the study design. IVs = instrumental variables, MR = Mendelian randomization, MR-PRESSO = MR pleiotropy residual sum and outlier, SNP = single-nucleotide polymorphisms.

An overview of our research design is shown in Figure 1. Our study consists of 5 parts: (1) identifying genetic variants as instruments for LST; (2) obtaining summarized data of tool single-nucleotide polymorphisms (SNPs) from GWAS of DR; (3) harmonizing exposure and outcome databases; (4) conducting MR analysis; (5) assessing MR analysis assumptions and sensitivity analyses. For the causal estimates from MR analysis to be valid, 3 key assumptions must be met: (1) the Relevance Criterion, meaning the genetic variants must be closely associated with the exposure; (2) the Independence Criterion, implying that the genetic variants should not be related to any potential confounders that might affect the exposure–outcome relationship; (3) the Exclusion–Restriction Criterion, meaning that the genetic variants affect the outcome only through the exposure. To ensure that the selected SNPs are not affected by potential confounders, all SNPs included in the study were checked using LDTrait (https://ldlink.nih.gov/?tab=ldtrait). Factors associated with a high risk of DR, including hyperglycemia, hypertension, dyslipidemia, and cataract surgery, were excluded. Finally, to ensure that the selected SNPs have strong predictive power, the corresponding F-statistic was calculated as F = (N − k − 1)/k × *R*^2^/(1 − *R*^2^), where N represents the sample size of the exposure data, k represents the number of IVs, and *R*^2^ represents the coefficient of determination.

### 2.3. IVs associated with LST

To elucidate the causal relationships of LST with DR, genetic tools were used summary data from the latest meta-analysis of a GWAS on LST. LST is defined based on survey questionnaires and self-reported durations of watching television, playing video games, and sitting in front of a computer. The specific measures depend on the contributing cohorts. A 1 standard deviation increase represents an additional 2.2 hours per day of screen time. This meta-analysis^[11]^ included as many as 703,901 individuals from 51 studies (94.0% European, 2.1% African, 0.8% East Asian, 1.3% South Asian, and 1.9% Hispanic). The report highlighted that 98 SNPs were significantly associated with LST (*P* < 5 × 10^‐9^, linkage disequilibrium *r*^2^ < 0.001). Through rigorous screening, representative SNPs were selected as IVs for data analysis. During the screening process, SNPs associated with risk factors for DR and palindrome sequences were excluded, including rs12992995, rs13107325, rs3791033, rs6010651, rs6857, and rs71658797. Ultimately, 63 SNPs were identified as IVs related to LST. In Table 1, we provide the effect sizes, standard errors, and *P*-values for each of the 63 SNPs associated with LST. The effect sizes range from −0.031 to 0.009, indicating varying degrees of influence on LST. The F-statistic values of the IVs ranged between 145.78 and 541.01, all of which were >10, indicating that these instruments have strong potential for predicting LST. Plenty of SNPs are located near genes involved in neural function and behavior regulation, supporting their potential role in influencing screen time behaviors (Table 1).

**Table 1:** Instrumental SNPs from GWAS on LST and DR.

SNP	EA	OA	Nearest gene	Exposure			Outcome			N	F
				Beta	SE	*P*-value	Beta	SE	*P*-value		
rs10189857	A	G	BCL11A	-0.027	0.004	7.8E-15	-0.012	0.014	.389	468924	252.33
rs10400776	A	C	VRK1	-0.026	0.004	3.45E-09	-0.025	0.017	.149	442658	182.69
rs10772643	T	C	EMP1	-0.039	0.006	5.88E-10	0.006	0.019	.770	468924	206.34
rs10889193	C	A	LINC01748	-0.024	0.004	4.94E-10	-0.007	0.015	.642	525479	200.33
rs11074658	T	C	GRIN2A	-0.024	0.004	9.21E-10	0.004	0.015	.799	442658	196.06
rs114590429	C	A	SCN2A	-0.038	0.006	3.03E-10	0.005	0.019	.799	468924	208.52
rs1160545	T	C	LINC01104	-0.029	0.004	1.14E-13	-0.017	0.015	.256	525490	284.96
rs12062845	C	A	DPYD	-0.028	0.004	2.41E-11	-0.007	0.016	.638	442658	186.96
rs12324720	A	G	HERC1	-0.027	0.005	3.53E-09	0.038	0.017	.026	521894	148.87
rs12617870	G	T	PCGEM1	-0.026	0.004	6.62E-14	-0.038	0.015	.010	524165	236.62
rs12678836	C	A	AC103409.1	-0.023	0.004	5.37E-11	-0.008	0.015	.591	442658	181.93
rs12962050	A	G	CELF4	-0.023	0.004	1.18E-10	-0.002	0.016	.913	468924	170.13
rs13235840	A	T	EXOC4	-0.031	0.005	3.48E-10	0.020	0.018	.269	468924	200.61
rs1375561	C	T	CADM2	-0.023	0.004	2.55E-10	-0.026	0.015	.071	525488	168.79
rs1391954	G	T	GRM5	-0.025	0.004	1.51E-09	0.008	0.014	.598	468924	217.28
rs144155998	TA	T	RNU6-692P	-0.043	0.006	1.3E-14	-0.019	0.019	.311	526370	332.04
rs148544378	T	C	RIT2	-0.088	0.014	6.55E-11	-0.123	0.109	.258	442658	224.21
rs17025214	T	C	HTR1F	-0.022	0.004	4.31E-09	0.019	0.016	.235	525491	145.78
rs17621391	C	T	MKRN1	-0.024	0.004	2.11E-09	-0.045	0.017	.007	442658	157.98
rs1860337	T	C	MARCHF10	-0.025	0.004	9.08E-11	-0.010	0.015	.497	525,485	212.09
rs1947066	G	A	GABRA6	-0.03	0.004	8.54E-12	-0.008	0.019	.656	442658	200.49
rs197439	A	G	INKA2	-0.026	0.004	3.29E-11	-0.026	0.014	.071	468924	228.28
rs1999065	C	T	TLR4	-0.025	0.004	1.24E-11	-0.014	0.015	.327	442658	196.93
rs2173650	T	G	TESC	-0.032	0.005	3.62E-09	-0.002	0.020	.918	468924	182.84
rs2229383	T	G	ILF3	-0.029	0.004	6.4E-13	-0.010	0.016	.543	468924	274.52
rs249960	G	A	ERAP1	-0.03	0.005	2.43E-09	-0.005	0.019	.788	468924	188.68
rs2529484	G	C	IMMP2L	-0.022	0.004	1.39E-09	0.005	0.015	.747	468,830	155.25
rs262890	A	G	HTR1A	-0.034	0.004	2.06E-16	-0.016	0.015	.279	468924	341.92
rs2783992	T	C	SMARCA2	-0.024	0.004	3.36E-10	0.003	0.015	.831	525491	201.55
rs2964252	A	G	LINC01470	-0.024	0.004	3.16E-10	-0.018	0.015	.241	468924	175.31
rs34864022	A	G	LINC01239	-0.048	0.008	4.71E-10	0.042	0.031	.168	521,893	200.00
rs36079846	T	C	VWC2L	-0.024	0.004	4.52E-10	-0.006	0.015	.658	469,809	202.42
rs3759344	G	A	MLF2	-0.045	0.006	4.26E-13	0.008	0.026	.748	484,638	270.26
rs396321	T	C	APC	-0.021	0.004	1.29E-09	-0.007	0.014	.652	442658	155.14
rs4303732	C	T	LINC01104	-0.027	0.004	5.37E-14	-0.017	0.015	.256	525491	246.80
rs4416502	G	A	ART3	-0.029	0.005	1.38E-09	-0.029	0.017	.089	468924	189.48
rs4483592	C	T	PACS1	-0.036	0.005	3.97E-12	0.006	0.019	.737	525491	249.01
rs4551799	A	G	FAM86C1	-0.024	0.004	6.91E-10	-0.013	0.016	.422	442658	183.04
rs558134	T	C	PHACTR1	-0.023	0.004	5.05E-10	-0.004	0.015	.782	468924	176.20
rs56151256	C	A	LINGO1	-0.029	0.004	1.17E-10	-0.034	0.018	.059	219,777	222.06
rs566017137	C	G	NR4A2	-0.066	0.011	1.23E-09	-0.044	0.040	.281	525479	212.88
rs62131183	A	G	SIX3-AS1	-0.054	0.009	3.31E-09	0.025	0.034	.471	525491	183.92
rs62151809	C	T	ZBTB46	-0.023	0.004	3.9E-09	0.010	0.014	.473	524,166	184.58
rs62244886	G	C	FOXP1	-0.024	0.004	1.24E-09	-0.021	0.015	.155	468924	194.01
rs6457816	T	C	PPARD	-0.041	0.007	3.97E-09	0.026	0.046	.572	468924	145.91
rs6685030	A	G	DNM3	-0.022	0.004	5.27E-10	-0.003	0.014	.810	468924	170.11
rs68049022	C	T	RPL17P35	-0.031	0.005	6.18E-11	-0.006	0.020	.763	243,769	218.15
rs73420302	C	G	CBX8	-0.03	0.005	3.04E-09	-0.007	0.016	.662	469,809	186.25
rs73581580	G	A	EXD3	-0.037	0.006	1.94E-10	0.028	0.018	.132	468924	209.41
rs7430216	C	T	LOC107986099	-0.025	0.004	2.5E-09	-0.009	0.017	.598	523,876	152.49
rs7432837	T	C	NSUN3	-0.024	0.004	3.24E-09	0.003	0.016	.849	468924	155.27
rs743699	A	G	RGS12	-0.027	0.004	1.18E-09	0.013	0.016	.433	442658	196.02
rs7613360	C	T	ACTBP13	-0.032	0.004	6.92E-16	-0.026	0.016	.103	468924	344.97
rs7615206	T	C	MST1R	-0.035	0.004	1.5E-22	-0.028	0.015	.064	442658	422.94
rs76267866	A	T	SAMSSON	-0.03	0.005	3.53E-10	-0.004	0.018	.842	468924	208.79
rs78140587	G	A	KRT8P5	-0.062	0.01	5.83E-10	0.012	0.040	.764	512,111	212.84
rs78394231	T	C	PDSS2	-0.038	0.007	3.53E-09	-0.036	0.023	.121	468924	179.74
rs7946119	C	T	NA	0.04	0.006	5.37E-11	-0.031	0.014	.032	482,490	541.01
rs7969719	C	T	MYO1H	-0.027	0.004	4.46E-13	0.005	0.017	.775	468924	220.75
rs841020	C	T	GPR26	-0.027	0.004	1.15E-09	-0.015	0.016	.344	442658	159.25
rs9513416	G	A	FARP1	-0.028	0.005	4.1E-09	-0.007	0.022	.737	243,411	147.62
rs9821299	G	A	CYP51A1P1	-0.029	0.005	3.63E-09	-0.027	0.018	.133	468924	178.56
rs9867121	A	C	ZBTB20	-0.032	0.005	2.02E-10	0.047	0.019	.015	468924	215.60

DR = diabetic retinopathy, EA = effect_allele, LST = leisure screen time, OA = other_allele.

### 2.4. GWAS summary data for DR

Data on DR was obtained from the Finnish database, DR is identified based on International Classification of Diseases, Revision 10 (H36.0). The control group for DR was defined as comprising individuals without DR and diabetic complications. The dataset included 10,413 patients with DR and 308,633 controls. Adjustment was made for age, gender, genetic relatedness, genotyping batch, and the first principal component. Summary data were obtained from the Finnish database, and data were extracted for each of the 69 SNPs related to LST. The data include the effect size and standard error of each SNP on DR.

### 2.5. GWAS summary data for risk factors of DR

To elucidate the mediating effect of LST through other risk factors on DR, the inverse variance weighted (IVW) method was employed to estimate the association between LST and other known risk factors for Dr. Recognized risk factors include hyperglycemia, hypertension, dyslipidemia, and cataract surgery.^[1]^ Summary data for these phenotypes were obtained from whole-genome association studies conducted by MAGIC,^[12,13]^ FinnGen,^[14]^ ICBP,^[15]^ GLGC,^[16]^ and the Neale Lab Consortium.^[17]^ Detailed information for all GWAS studies included in our research is presented in Table 2.

**Table 2:** Details of the GWASs included in the MR.

Phenotype	Consortium	Participants	Web source
Leisure screen time	A meta-analysis of GWAS	703,901	https://pubmed.ncbi.nlm.nih.gov/36071172/
Diabetic retinopathy	FinnGen	314,042	https://www.finngen.fi/en/access_results
HbA1c	MAGIC	46,368	https://gwas.mrcieu.ac.uk/
Hypertension	FinnGen	314,042	https://www.finngen.fi/en/access_results
SBP	ICBP	757,601	https://www.ncbi.nlm.nih.gov/
DBP	ICBP	757,601	https://www.ncbi.nlm.nih.gov/
HDL-C	GLGC	187,167	http://csg.sph.umich.edu/willer/public/lipids2013/
LDL-C	GLGC	173,082	http://csg.sph.umich.edu/willer/public/lipids2013/
Triglycerides	GLGC	177,861	http://csg.sph.umich.edu/willer/public/lipids2013/
Total cholesterol	GLGC	187,365	http://csg.sph.umich.edu/willer/public/lipids2013/
Cataract surgery	Neale Lab Consortium	5799	http://www.nealelab.is/uk-biobank

DBP = diastolic blood pressure, GLGC = Global Lipids Genetics Consortium, HbA1c = hemoglobin A1c, HDL-C = high-density lipoprotein cholesterol, ICBP = International Consortium for Blood Pressure, LDL-C = low-density lipoprotein cholesterol, MAGIC = Meta-analysis of Glucose and Insulin Related Traits Consortium, SBP = systolic blood pressure.

### 2.6. Statistical analysis

In this study, MR analyses were conducted using R software version 4.2.2. Various MR techniques were employed to assess the relationship between LST and DR, this included IVW, weighted median, and MR-Egger methods. The IVW method.^[18]^ calculates the inverse variance-weighted average of ratio estimates from 2 or more genetic instruments. This method assumes that the IVs influence the outcome solely via the exposure under study. The rationale for using IVW lies in its efficiency in providing precise estimates when all IVs are valid and there is no horizontal pleiotropy. IVW is particularly effective in integrating multiple SNPs to derive a comprehensive estimate of the causal effect.

The weighted median method offers robust causal effect estimates even when up to 50% of the genetic instruments are invalid. This method is advantageous in scenarios where some instruments might be influenced by pleiotropic effects, ensuring that the median estimate remains unbiased. The MR-Egger method can detect and adjust for directional pleiotropy, where the IVs have direct effects on the outcome independent of the exposure. This method provides an intercept term that indicates the presence of pleiotropy, thereby enhancing the reliability of causal inference. The simultaneous use of IVW, weighted median, and MR-Egger methods ensures robust and reliable causal estimates by leveraging the strengths of each approach while mitigating their individual limitations.

Sensitivity analysis played a crucial role in this research, particularly in scenarios where pleiotropy could distort MR estimates. The Cochran *Q* statistic was used to assess the heterogeneity of the IVW method (*P* < .05 indicating the presence of pleiotropy), while the MR-Egger regression intercept was utilized to detect directional pleiotropy (*P* < .05 indicating presence). To address potential horizontal pleiotropy and ensure the robustness of our results, additional sensitivity analyses were conducted using the MR pleiotropy residual sum and outlier (MR-PRESSO) method. MR-PRESSO detects and corrects for pleiotropy by identifying outliers and providing corrected estimates. This method enhances the reliability of causal inference by accounting for potential biases due to pleiotropy. Furthermore, MR-leave-one-out analysis was conducted to determine if individual SNPs significantly influenced MR estimates.

## 3. Results

### 3.1. The causal relationship between LST and DR

**Figure 2.**
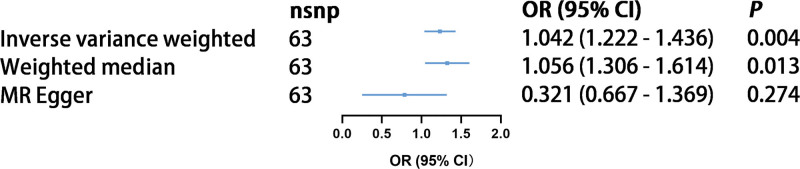
Mendelian randomization estimates of leisure screen time on the risk of diabetic retinopathy.

To explore the causal relationship between LST and DR, 3 different MR analysis methods were employed. The methods used included IVW, weighted median, and MREgger. Using the IVW method with 63 SNPs associated with LST, we found strong evidence of a potential causal effect of LST on DR, with significant statistical significance (OR = 1.22, 95% CI = 1.04–1.43, *P* = 1.38 × 10^‐2^). Similarly, using the weighted median method, we obtained a comparable risk estimate (OR = 1.30, 95% CI = 1.05–1.61, *P* = 1.46 × 10^‐2)^. However, the MR-Egger method did not find a significant effect (OR = 0.66, 95% CI = 0.32–1.36, *P* = .273), suggesting potential pleiotropy (see Fig. 2). The pleiotropy test (intercept = 0.017; standard error = 0.01, *P* = .09) further indicated possible biases in the IV estimates. To strengthen the analysis and address potential pleiotropy, we conducted additional sensitivity analyses using the MR-PRESSO method. The MR-PRESSO global test for heterogeneity did not indicate significant pleiotropy (*P*-value > 0.05), and no significant outliers were detected. These findings are consistent with the Cochran *Q* test results, which showed no heterogeneity (IVW *P*-value = 0.170; MR-Egger *P*-value = 0.128). Additionally, the funnel plot was symmetrical, indicating no heterogeneity (Figure S1, Supplemental Digital Content, http://links.lww.com/MD/N768). The MR regression slopes and individual causal estimates for these 63 SNPs are shown in Figure S2 Supplemental Digital Content, http://links.lww.com/MD/N768 and Figure S3, Supplemental Digital Content, http://links.lww.com/MD/N768. In the leave-one-out sensitivity analysis, no single SNP significantly altered the overall effect of LST on DR, demonstrating the robustness of the results (Figure S4, Supplemental Digital Content, http://links.lww.com/MD/N768).

### 3.2. The causal effects of LST on risk factors for DR

Following the harmonization of LST and DR risk factor data, we excluded palindrome SNPs with moderate allele frequencies. Genetic variations found to be associated with potential confounders were also excluded from the analysis. Furthermore, to ensure the reliability of these IVs, F-statistic values were individually computed, all of which yielded values >10, demonstrating the credibility of the selected IVs. We used the IVW method to study the relationship between LST and several risk factors for Dr. The study results showed a causal impact of LST on hypertension, while no causal effects of LST were observed on other potential risk factors for DR (see Table 3).

**Table 3:** MR estimates of the associations from LST on common risk factors.

Outcome	Causal effect (95% CI)	*P*-value
HbA1c	1.01 (0.95–1.08)	.56
Hypertension	1.21 (1.11–1.33)	2.80 × 10^-5^
SBP	0.86 (0.37–2.03)	.74
DBP	1.09 (0.67–1.76)	.72
HDL-C	1.06 (0.83–1.35)	.61
LDL-C	0.84 (0.56–1.25)	.40
Triglycerides	0.98 (0.83–1.15)	.82
Total cholesterol	0.89 (0.62–1.12)	.53
Cataract surgery	0.99 (0.92–1.06)	.86

DBP = diastolic blood pressure, HbA1c = hemoglobin A1c, HDL-C = high-density lipoprotein cholesterol, LDL-C = low-density lipoprotein cholesterol, SBP = systolic blood pressure.

### 3.3. Calculation of power

In this study, the power corresponding to LST was calculated using an online tool (https://shiny.cnsgenomics.com/mRnd/). The relevant parameters were set as follows: Type I error rate of 0.05, *R*^2^ of 0.019, and based on the total sample size for DR, resulting in a power of 86%. This outcome indicates that the study has sufficient power to investigate the causal relationship between LST and Dr.

## 4. Discussion

In this study, the relationship between LST and DR was investigated using MR methods. This approach utilizes genetic variation as IVs to overcome limitations in establishing causal relationships inherent in traditional research methodologies. Through rigorous screening, 63 SNPs were identified as IVs related to LST. Many of these SNPs are located in or near genes involved in neurological processes and behavioral traits. rs10772643 is located near EMP1, a gene involved in cell proliferation and differentiation, including neuronal cells. EMP1 has been implicated in neurogenesis and neuronal differentiation, suggesting its role in influencing behaviors related to screen usage.^[19]^ Similarly, rs12962050 is near CELF4, a gene associated with RNA binding and regulation of gene expression in the brain. CELF4 is known to affect synaptic function and neuronal excitability, which may impact cognitive functions and behaviors related to screen time.^[20]^ The involvement of CELF4 in cognitive processes provides a plausible mechanism for its influence on LST. Further, rs1947066 is near GABRA6, which encodes a subunit of the GABA-A receptor. This receptor is critical for inhibitory neurotransmission in the brain and may affect screen-related behavior and usage patterns. Studies have indicated that variations in GABRA6 are linked to anxiety and stress responses, which could indirectly influence screen time behaviors.^[21]^ These examples indicate that the SNPs selected in this study have a plausible biological mechanism for their impact on LST.

We observed significant differences in the results obtained from different MR methods used to evaluate the causal relationship between LST and Dr. The IVW method and the weighted median method both suggested a significant causal effect of LST on DR (IVW OR: 1.22, 95% CI: 1.04–1.43, *P* = 1.38 × 10^‐2^; weighted median OR: 1.30, 95% CI: 1.05–1.61, *P* = 1.46 × 10^‐2^). However, the MR-Egger method did not find a significant effect (OR: 0.66, 95% CI: 0.32–1.36, *P* = .273). The MR-Egger method is specifically designed to detect and adjust for horizontal pleiotropy, which occurs when genetic variants affect the outcome through pathways other than the exposure of interest. The presence of horizontal pleiotropy can bias causal estimates in MR analyses. The MR-Egger intercept test provides an indication of pleiotropy; in this study, the intercept was not significantly different from zero (*P* = .09), suggesting potential pleiotropy but not definitively confirming it. The divergent results between IVW, weighted median, and MR-Egger methods can be attributed to their underlying assumptions and sensitivities to pleiotropy: IVW method: assumes no horizontal pleiotropy and that all genetic instruments are valid. This method is highly efficient and provides precise estimates when these assumptions hold true. However, if any of the instruments are pleiotropic, the estimates can be biased. Weighted median method: provides robust causal estimates even if up to 50% of the instruments are invalid or pleiotropic. This robustness makes it reliable under weaker assumptions but potentially less efficient than IVW when all instruments are valid. MR-Egger method: detects and adjusts for horizontal pleiotropy. It provides an unbiased estimate of the causal effect even in the presence of pleiotropy. However, MR-Egger is less powerful and more susceptible to weak instrument bias, which may explain the lack of significant findings in this study.

The current study indicates that with the proliferation of smart devices, individuals are increasingly immersed in screens, whether for work or leisure.^[22]^ Prolonged screen time, especially during leisure, is closely associated with sedentary behavior and reduced physical activity, lifestyle changes directly linked to increased risks of diabetes and its complications.^[23,24]^ Dirani et al’s study^[25]^ suggested physical inactivity as a risk factor for DR, a finding further corroborated by Ren et al^[26]^ in a systematic review and meta-analysis, where physical activity was associated with a lower risk of Dr. However, due to the limitations of observational studies, these findings are susceptible to unobserved confounders or reverse causality. This study, for the first time, confirms the association between LST and DR through MR, mitigating residual confounding by utilizing genetic variation as IVs. This enables a robust estimation of the association between LST and the risk of Dr.

Using the IVW method, we found that LST significantly increased the risk of hypertension. This finding is consistent with previous research indicating a link between prolonged screen use and reduced physical activity associated with hypertension.^[26]^ Importantly, this study did not observe a direct causal effect of LST on other known risk factors for DR, such as blood glucose levels and dyslipidemia. This may suggest that the influence of LST on DR risk primarily operates through other nonmetabolic pathways, such as lifestyle changes and reduced physical activity, rather than directly affecting these biomarkers.

The association between LST and the increased risk of DR may involve complex biological mechanisms. Firstly, hypertension, a key risk factor for DR, can exacerbate retinal microvascular damage by increasing retinal vascular pressure, impairing endothelial function, and reducing blood circulation efficiency. Secondly, inflammation and oxidative stress play critical roles in the development of Dr. Sedentary behavior has been associated with increased levels of inflammation and oxidative stress.^[27,28]^ Oxidative stress can directly damage retinal cells by activating reactive oxygen species and other reactive metabolites,^[29,30]^ accelerating pathological changes in retinal microvasculature. Lastly, changes in retinal blood flow represent another important mechanism linking LST and DR risk. Studies have shown that increased physical activity can improve microvascular function and increase retinal blood flow, contributing to maintaining retinal health.^[31,32]^ Conversely, LST, by reducing physical activity, may indirectly lead to decreased retinal microvascular function, affecting retinal oxygen and nutrient supply, further increasing the risk of Dr.

Given these findings, several public health recommendations are advised. First, reducing LST is essential, especially for individuals with diabetes. Public health campaigns and educational programs should highlight the risks associated with prolonged screen time and promote strategies such as the 20–20–20 rule (taking a 20-second break to look at something 20 feet away every 20 minutes) to encourage regular breaks during screen activities, thereby reducing continuous sedentary behavior. Encouraging regular physical activity is also crucial to counteract the effects of sedentary behavior. This can help manage body weight, improve cardiovascular health, and reduce hypertension, which subsequently lowers the risk of Dr. Healthcare providers should advocate for structured exercise programs tailored to individuals with diabetes, focusing on reducing sedentary time and enhancing overall health. Moreover, regular blood pressure monitoring is vital for individuals at risk of Dr. Early detection and management of hypertension can significantly mitigate the risk of developing Dr. Implementing strategies to manage hypertension, including dietary modifications, increased physical activity, and, if necessary, medication, is crucial given the significant link between LST and increased blood pressure found in our study.

Although the MR method has advantages in reducing the influence of confounding factors, its results are still limited by the selection of genetic variants used. One limitation of our study is the primary use of data from populations with European ancestry. This focus may restrict the generalizability of our findings to other ethnic groups. Genetic variations and environmental factors can differ significantly across populations, potentially influencing the relationship between LST and Dr. As a result, the causal links identified in this study may not fully apply to individuals of non-European descent. To enhance the robustness and applicability of our findings, further research is needed involving more ethnically diverse cohorts. Studies including participants from various genetic backgrounds can help validate our results and provide a more comprehensive understanding of how LST influences DR risk across different populations. Such research will also contribute to developing tailored public health strategies that consider the genetic and environmental diversity of global populations. In conclusion, while our study provides valuable insights into the relationship between LST and DR, the generalizability of these findings is limited by the homogeneity of the study population. Future studies should aim to include more diverse populations to ensure the broad applicability of the results and to develop more inclusive and effective public health recommendations. Furthermore, further research should investigate the specific impacts of different types of screen usage habits (such as work and leisure) on the risk of DR and explore possible biological mechanisms. While the MR method reduces the impact of confounding factors, the intervention of unknown factors is still inevitable.

In summary, the findings of this study emphasize the importance of managing screen time responsibly in daily life. By reducing LST, both directly and indirectly, the risk of DR can be reduced, offering a new perspective for public health interventions and personal health management. However, to understand this complex relationship more deeply and develop effective intervention strategies, further research and exploration are needed.

## 5. Conclusion

This is the first MR study to explore the causality from LST on DR. Our MR analysis support the hypothesis that LST could increase DR incidence.

## Acknowledgements

We want to acknowledge the participants and investigators of the FinnGen study.

## Author contributions

**Conceptualization:** Jun-Jie Liu.

**Data curation:** Yuan-Yuan Tang, Hong-Jing Gu, Xiao-Shu Wang.

**Formal analysis:** Yuan-Yuan Tang.

**Funding acquisition:** Jun-Jie Liu.

**Methodology:** Hong-Jing Gu, Xiao-Shu Wang.

**Supervision:** Yuan-Yuan Tang, Jun-Jie Liu.

**Visualization:** Yuan-Yuan Tang, Jun-Jie Liu.

**Writing – original draft:** Yuan-Yuan Tang, Jun-Jie Liu.

**Writing – review & editing:** Chun-Mei Tan.

## Supplementary Material


