# Supplementary material for: Leisure screen time and diabetic retinopathy risk: A Mendelian randomization study
Source: Medicine (Baltimore). 2024 Oct 25;103(43):e40099. doi: 10.1097/MD.0000000000040099 (PMC11521079; doi:10.1097/MD.0000000000040099)
Supplement: Supplementary file 1 [file medi-103-e40099-s001.pdf]

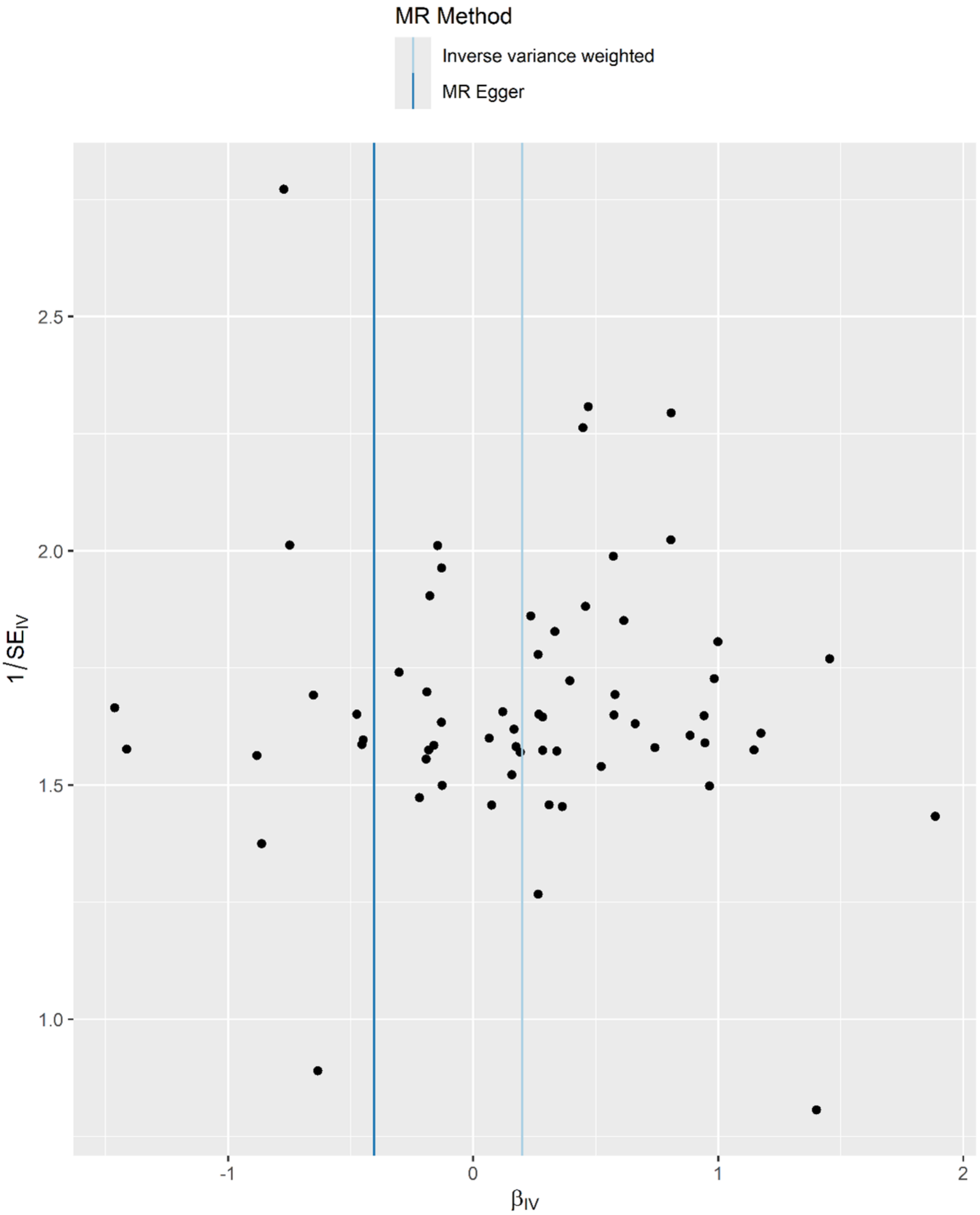

Supplementary Figure 1 Mendelian Randomization Funnel Plot for the Impact of Leisure Screen Time on the Risk of Diabetic Retinopathy.

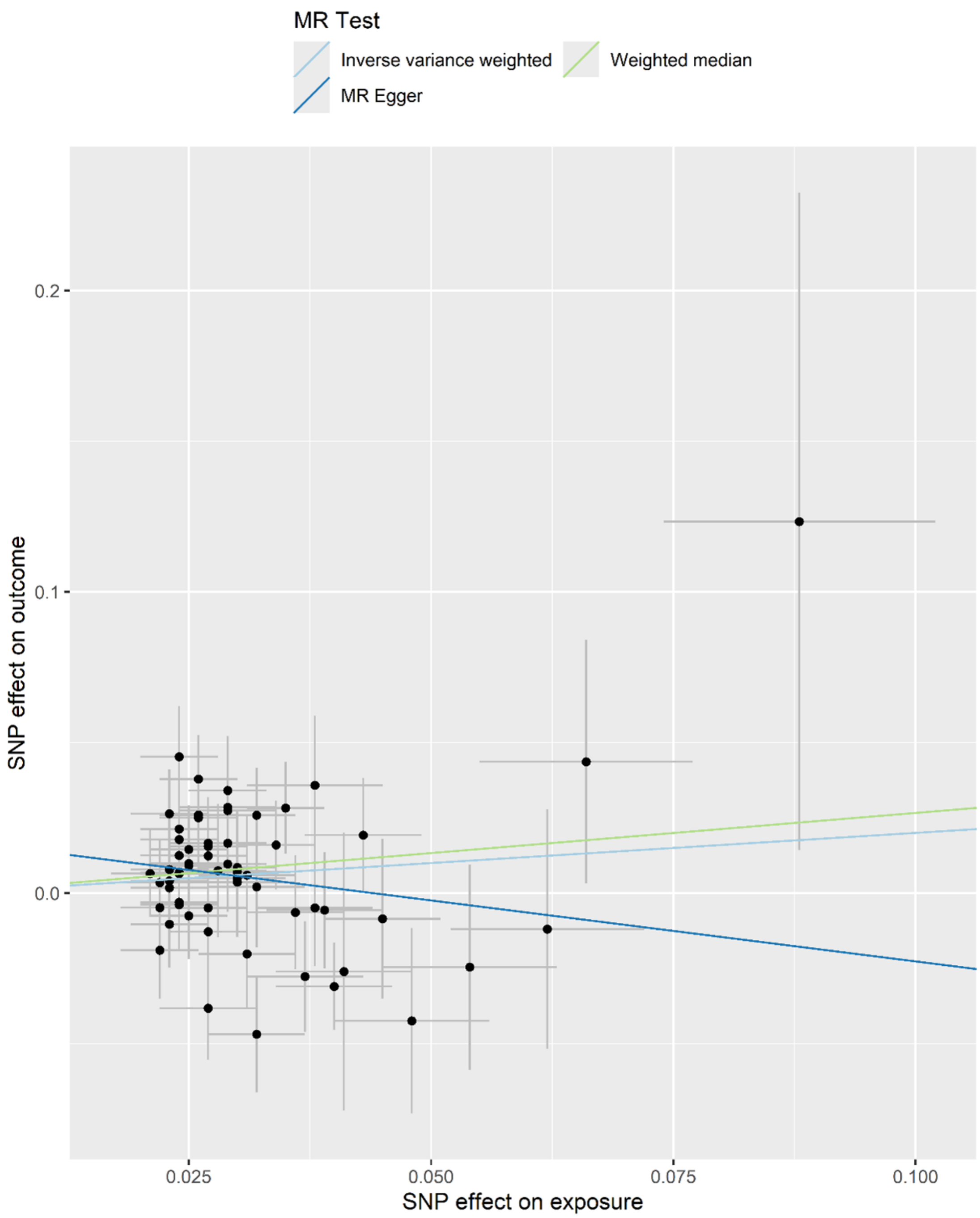

Supplementary Figure 2 Scatter Plot for the Impact of Leisure Screen Time on the Risk of Diabetic Retinopathy.

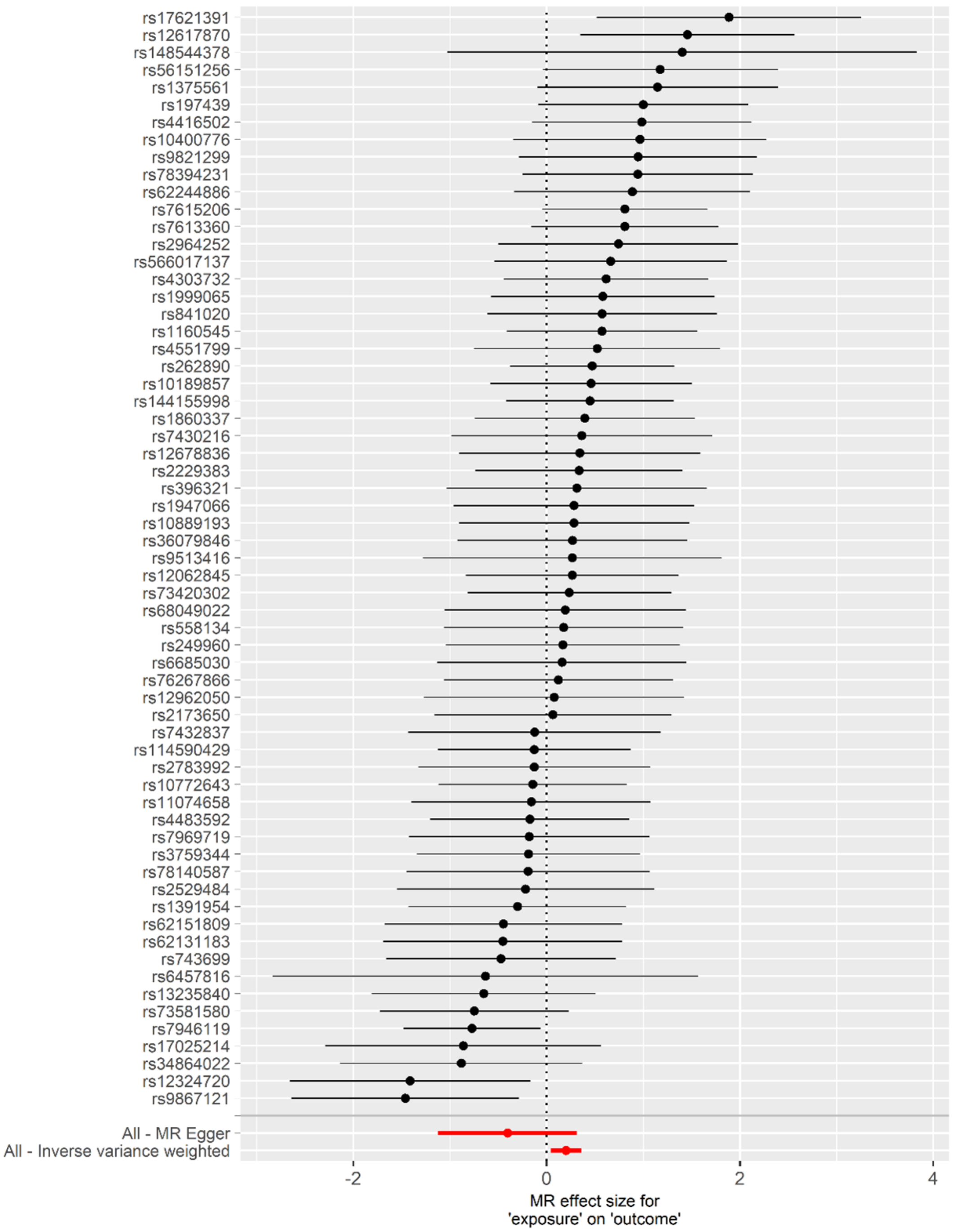

Supplementary Figure 3 Forest Plot of SNP Effect Estimates for the Impact of Leisure Screen Time on the Risk of Diabetic Retinopathy.

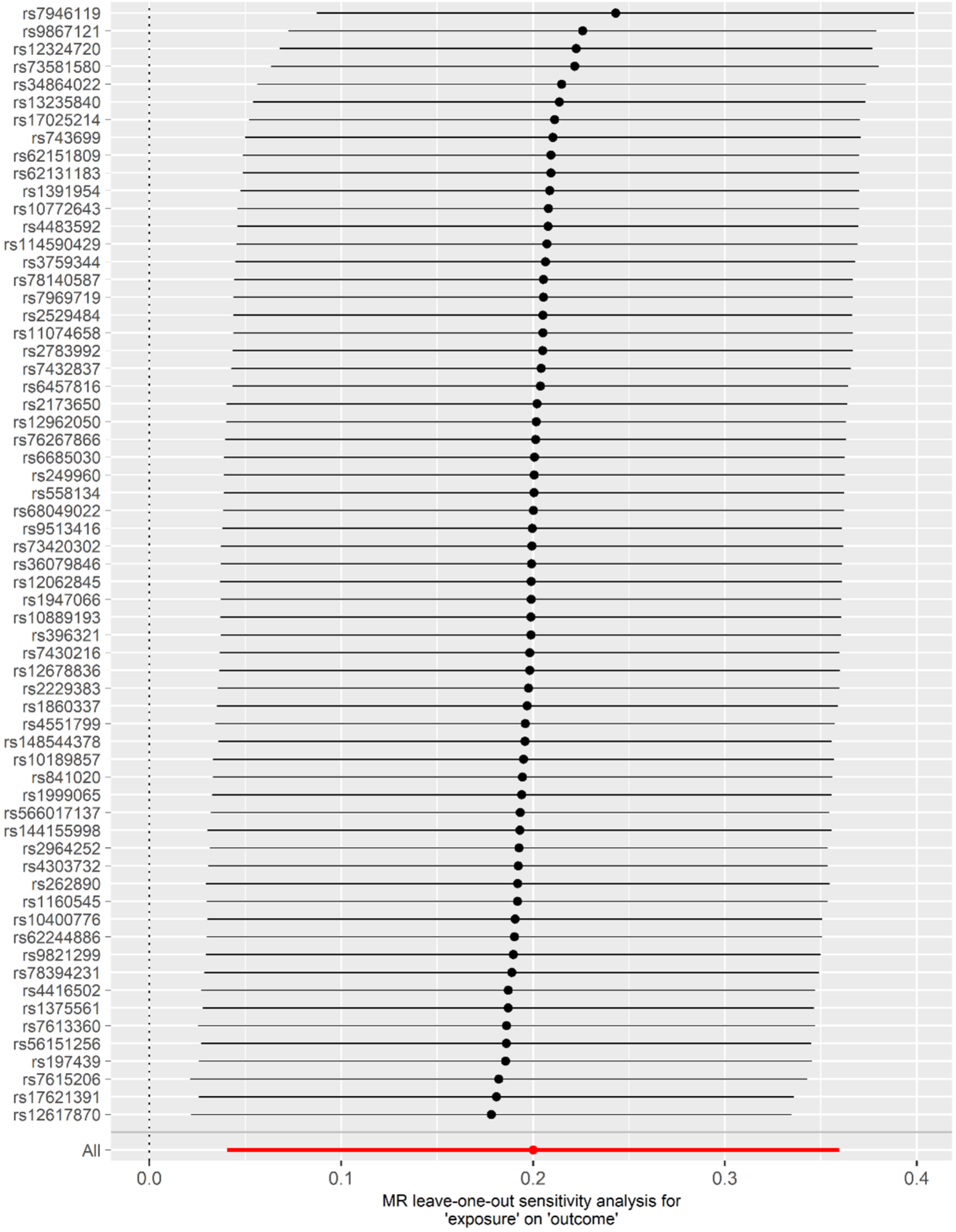

Supplementary Figure 4 Leave-One-Out Sensitivity Analysis: Impact of Leisure Screen Time on the Risk of Diabetic Retinopathy
